# Anti-tumor effect of estrogen-related receptor alpha knockdown on uterine endometrial cancer

**DOI:** 10.18632/oncotarget.9151

**Published:** 2016-05-03

**Authors:** Hiroshi Matsushima, Taisuke Mori, Fumitake Ito, Takuro Yamamoto, Makoto Akiyama, Tetsuya Kokabu, Kaori Yoriki, Shiori Umemura, Kyoko Akashi, Jo Kitawaki

**Affiliations:** ^1^ Department of Obstetrics and Gynecology, Kyoto Prefectural University of Medicine, Graduate School of Medical Science, Kajii-cho, Kamigyo-ku, Kyoto, Japan

**Keywords:** apoptosis, cell cycle arrest, ERRα, VEGF, uterine endometrial cancer

## Abstract

Estrogen-related receptor (ERR)α presents structural similarities with estrogen receptor (ER)α. However, it is an orphan receptor not binding to naturally occurring estrogens. This study was designed to investigate the role of ERRα in endometrial cancer progression. Immunohistochemistry analysis on 50 specimens from patients with endometrial cancer showed that ERRα was expressed in all examined tissues and the elevated expression levels of ERRα were associated with advanced clinical stages and serous histological type (*p* < 0.01 for each). ERRα knockdown with siRNA suppressed angiogenesis *via* VEGF and cell proliferation *in vitro* (*p* < 0.01). Cell cycle and apoptosis assays using flow cytometry and western blot revealed that ERRα knockdown induced cell cycle arrest during the mitotic phase followed by apoptosis initiated by caspase-3. Additionally, ERRα knockdown sensitized cells to paclitaxel. A significant reduction of tumor growth and angiogenesis was also observed in ERRα knockdown xenografts (*p* < 0.01). These findings indicate that ERRα may serve as a novel molecular target for the treatment of endometrial cancer.

## INTRODUCTION

Endometrial cancer is the fourth most common malignancy among women [1]. In 2012, 53,630 new cases and 8,590 deaths due to endometrial cancer were reported in the United States [2]. The incidence has doubled in the last decade, especially in Japan. Most cases diagnosed at an early stage and lower histological grade can be cured. However, the prognosis of patients with higher histological grade and invasion beyond the uterus is poor [3]. Other therapeutic modalities such as chemotherapy and radiation therapy are not well-established since the tumor is less sensitive to these [4]. To date, despite the recent advances in molecular targeted therapy, only few effective therapies are available. Therefore, novel treatment strategies for endometrial cancer are desired.

The main characteristic of uterine endometrial cancer is that it is hormone dependent [5]. Estrogen is known to be associated with carcinogenesis and to promote the progression of endometrial cancer [6]. To inhibit the progression of breast cancer, the representative estrogen-dependent disease, an antagonist for estrogen receptor (ER) and/or inhibitors of aromatase, the enzyme involved in estrogen synthesis, have been widely used [7, 8]. However, these have not been very effective for uterine endometrial cancer, suggesting the existence of more complicated estrogen signaling pathways in this disease.

Estrogen-related receptor (ERR)α, one of the orphan members of the nuclear receptor superfamily, is a constitutively active receptor that shares considerable structural homology with the classical ERα and ERβ [9, 10]. Unlike ERs, ERRα does not bind to naturally occurring estrogens, but shares other biochemical activities with ERs, including binding to ERE. ERRs also bind to the half-site ERE, 5′-AGGTCA-3′, known to as estrogen-related responsive element (ERRE) [11–13]. ERRα is abundantly expressed in tissues rich in mitochondria and with high energy demands such as the heart, brain, and kidneys and controls the cellular energy metabolism [14, 15]. The expression of the three ERR isoforms has been identified in various types of cancer, including breast, prostate, colon, uterine endometrium, and ovarian cancer [16–20]. In particular, increased ERRα levels are correlated with a higher risk of recurrence and poor prognosis in breast cancer [16]. However, the expression and function of ERRα in endometrial cancer remains unclear.

We first hypothesized that a more complicated crosstalk exists between ERα and ERRα. We previously reported that ERRα competed with ERα for ERE binding and inhibited ERα transcriptional activity in ERα positive endometrial cancer cells [19]. In this study, to elucidate the function of ERRα in endometrial cancer without ERα interference, we performed loss of function experiments using ER-negative cell lines. We demonstrated various anti-tumor effects of ERRα knockdown and potential of ERRα as a target for molecular therapy for uterine endometrial cancer.

## RESULTS

### ERRα expression in uterine endometrial cancer cells and its link to prognosis

**Figure 1:**
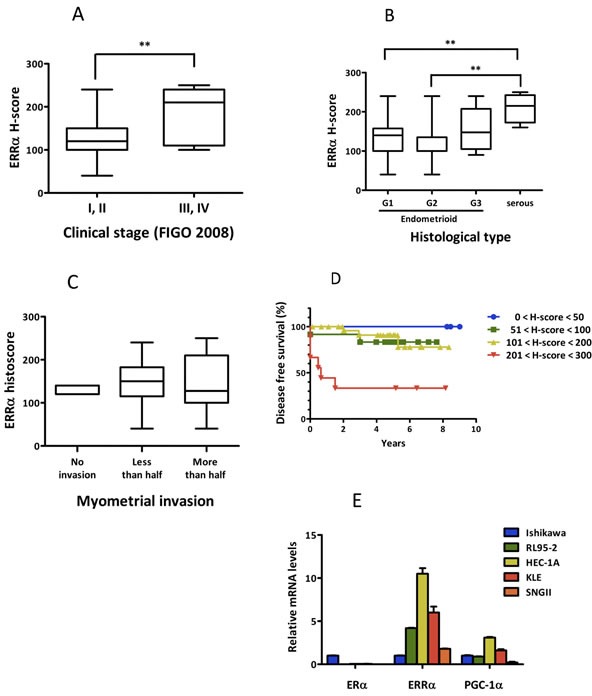
ERRα expression in uterine endometrial cancer *in vitro* and *in vivo* **A.**-**C.**, Association of ERRα expression with clinico-pathological factors. ERRα expression levels examined by immunohistochemistry were evaluated by H-score and their association with clinical parameters, including clinical stage based on FIGO 2008 classification **A.**, histopathological type **B.**, and myometrial invasion **C.** are presented. Statistical significance was determined by using the Kruskal-Wallis H-test. The Mann-Whitney U-test with Bonferroni correction was used as a post hoc test. **D.**, Kaplan-Meier analysis of disease free survival in 50 patients with endometrial cancer is shown in association with ERRα expression (*P* = 0.03). *P* values were based on log rank test. **E.**, The expression of ERα, ERRα, and PGC-1α, known as a co-activator of ERRα, in endometrial cancer cells was examined by real-time PCR. Significant differences are indicated as ** for *P* < 0.01 and * for *P* < 0.05 between groups.

We first performed an immunohistological analysis using tissue specimens. Patient characteristics are shown in Table 1. Of the 53 patients, specimens from 50 cases were available for the analysis. ERRα was expressed in all tissue specimens examined. The H-score distribution of ERRα is presented in Table 2. ERRα expression was significantly elevated at higher clinical stages and in serous adenocarcinoma when compared with endometrioid adenocarcinoma (Figure 1A, 1B). However, there was no correlation between ERRα expression and the tumor histological grade and the depth of uterine myometrial invasion (Figure 1B, 1C). The expression of ERα was examined by immunohistochemistry, but there was no association between ERα and ERRα (data not shown). The Kaplan-Meier analysis indicated that the level of ERRα expression was inversely correlated with disease free survival (Figure 1D), suggesting that ERRα could be an independent prognostic factor for uterine endometrial cancer. We next performed real-time PCR to elucidate the expression levels of ERα, ERRα, and PGC-1α, known as a robust co-activator of ERRα, in uterine endometrial cancer cell lines. All cell lines expressed ERRα and PGC-1α regardless of ERα expression, showing high expression in HEC-1A and KLE cells (Figure 1E).

**Table 1:** Patient characteristics

Age (years)	28-80 (mean: 56.1)
**Histological type**	
Endometrioid	
Grade1 Grade2 Grade3 Serous and others	28 (56.0%) 8 (16.0%) 8 (16.0%) 6 (12.0%)
**Myometrial invasion**	
No invasion Less than half More than half Not known	6 (12.0%) 22 (44.0%) 20 (20%) 2 (2%)
**Lymph node metastasis**	
Negative Positive	44 (88.0%) 6 (12.0%)
**Clinical stage (FIGO 2008)**	
I II III IV	34 (68.0%) 5 (10.0%) 7 (14.0%) 4 (8.0%)
**Total**	50 (100%)

**Table 2:** Distribution of ERRα-H-score

H-score	Patient number (%)
1-50 51-100 101-200 201-300	3 (6.0%) 12 (24.0%) 26 (52.0%) 9 (18.0%)
Total	50 (100%)

### Effect of overexpression and knockdown of ERRα on angiogenesis in endometrial cancer cells

**Figure 2:**
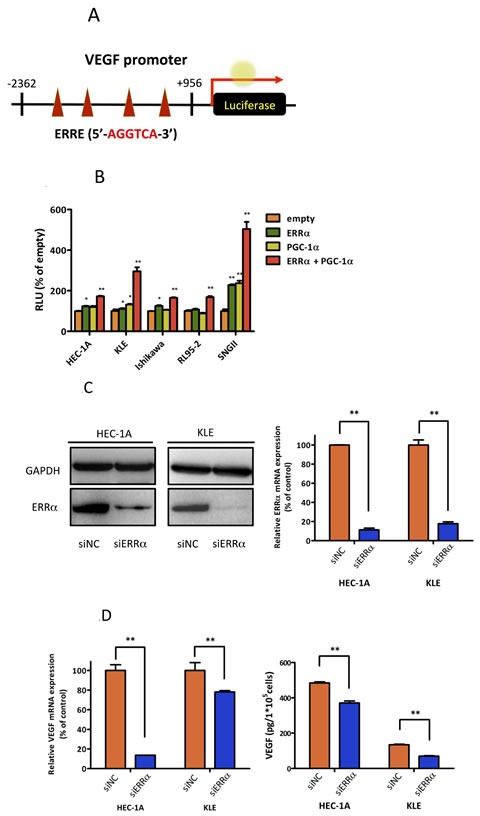

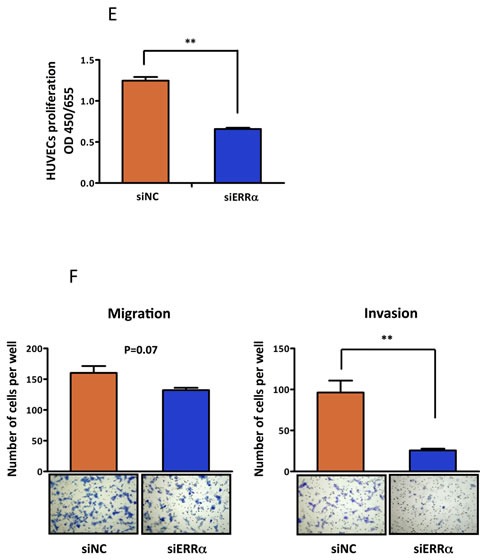
Effect of ERRα knockdown on VEGF expression and angiogenesis **A.**, VEGF promoter-luciferase reporter containing four copies of the ERRE site, (5′-AGGTCA-3′) was transfected into endometrial cancer cell lines. **B.**, Luciferase transcriptional activity using the VEGF promoter reporter. The pcDNA3.1-ERRα and PGC-1α plasmids were transiently transfected into HEC-1A and KLE cells using Lipofectamine LTX. The pcDNA3.1-empty vector was used as a negative control. After transfection, the cells were incubated for 24 h and then lysed. The promoter activities were assessed by dual 2 2luciferase assay. Data represent means ± SEM (*n* = 3). **C.**, The effect of ERRα knockdown was evaluated by real-time PCR and western blot analysis. Data represent means ± SEM (*n* = 3). **D.**, VEGF expression was assessed by real-time PCR and ELISA in HEC-1A and KLE cells transfected with the negative control (siNC) and ERRα (siERRα). After the transfection, the cells were incubated for 48 h and then lysed for real-time PCR and incubated for 72 h for ELISA. Data represent means ± SEM (real-time PCR;*n* = 3, ELISA; *n* = 4). **E.**, HUVEC proliferation assay was performed to evaluate the angiogenic potential of cancer cells. HUVECs were cultured with the collected supernatants from endometrial cancer cells transfected with siRNA. After 72 h incubation, the proliferation of HUVECs was measured using the WST-8 assay. Data represent means ± SEM (*n* = 4). **F.**, Matrigel invasion assay. HUVEC were co-cultured with cancer cells transfected with siRNA in a double-chamber system. After 6 h incubation, migration and invasion of HUVECs were assessed. Cells were counted in three high-power fields (× 200). Data represent means ± SEM (*n* = 3). Significant differences are indicated as ** for *P* < 0.01 and * for *P* < 0.05. Statistical analyses were performed using the two-tailed Student's t test. Each assay was repeated at least three times. HUVEC, human umbilical vein endothelial cell; RLU, relative luciferase unit.

Angiogenesis is an important factor for tumor progression. VEGF is a prominent factor for tumor angiogenesis. VEGF includes four 5′-AGGTCA-3′ sites, which are known to be ERRα binding sites [21], in its promoter region (Figure 2A). Therefore, we performed luciferase assays using a reporter containing the *VEGF* promoter region. Our results showed that ERRα transactivated VEGF. In addition, ERRα synergistically increased VEGF promoter activity in the presence of PGC-1α in all uterine endometrial cell lines (Figure 2B). To understand the detailed molecular mechanism of ERRα in endometrial cancer, we next performed loss of function experiments using siRNAs. We selected HEC-1A and KLE cell lines, which are negative for ERα and naturally express high levels of ERRα and PGC-1α (Figure 1E). ERRα knockdown with siRNA in both cell lines was confirmed by real-time PCR and western blot analysis (Figure 2C). VEGF expression at the mRNA and protein levels was significantly reduced in cells knocked down for ERRα (Figure 2D). Additionally, HUVECs were used to assess the effects of ERRα knockdown on endothelial cells [22]. ERRα knockdown significantly suppressed HUVEC proliferation (Figure 2E). Our invasion experiments also revealed that ERRα knockdown significantly suppressed cell invasion and tended to decrease HUVEC migration (Figure 2F).

### Effect of ERRα knockdown on cell growth and its association with phases of the cell cycle and apoptosis in endometrial cancer cells

**Figure 3:**
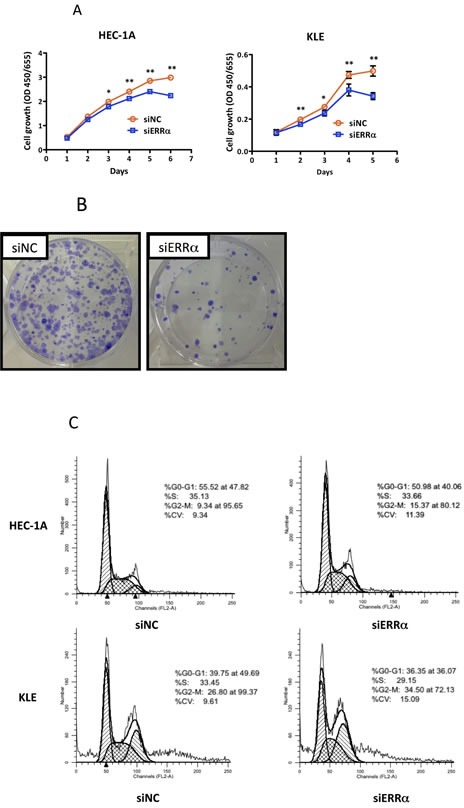

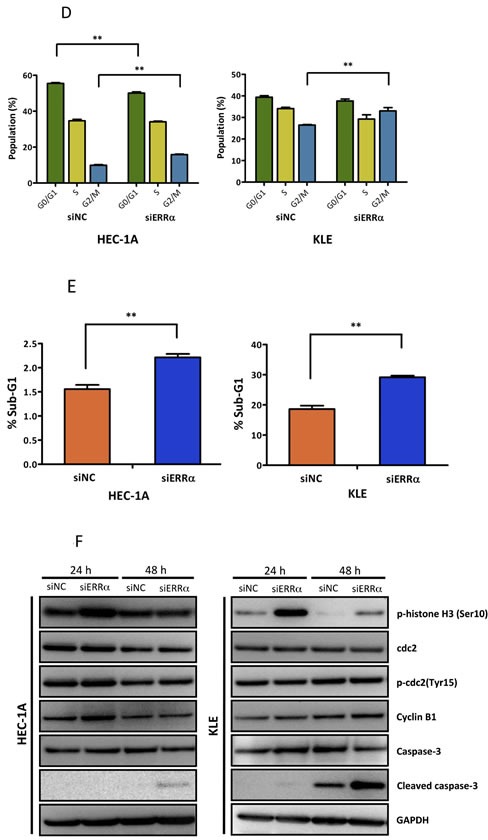

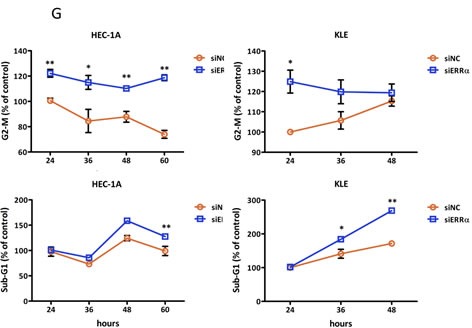
Effect of ERRα knockdown on the proliferation of endometrial cancer cells **A.**, WST-8 cell proliferation assay. After siRNA transfection, the viability of cancer cells was determined every 24 h. Data represent means ± SEM (*n* = 4). **B.**, Colony formation assay. Twenty-four hours after siRNA transfection, cancer cells were reseeded at a density of 3 3200 cells/well in 6-well plates and cultured for 14 days. Cells were then stained with crystal violet. **C.**, Cell cycle analysis using flow cytometry. Twenty-four to 60 h after siRNA transfection, cancer cells were collected and flow cytometry analysis was performed. **D.**, Cell distribution in each phase of the cell cycle. Data represent means ± SEM (*n* = 3). **E.**, Sub-G1 population. Data represent means ± SEM (*n* = 3). **F.**, Western blot analysis of proteins involved in the G2/M phase and apoptosis. Twenty-four to 48 h after siRNA transfection, western blotting analysis for phospho-histone H3 (Ser10), CDC2, phospho-CDC2 (Tyr15), cyclin B1, and cleaved caspase-3 was performed using cell lysates. GAPDH was used as a loading control. **G.**, Time-course analysis of the percentage of cells in G2/M and sub-G1 phases. Data represent means ± SEM (*n* = 3). Significant differences are indicated as ** for *P* < 0.01 and * for *P* < 0.05. Each assay was repeated at least three times. siNC, negative control siRNA; siERRα, ERRα siRNA.

To examine the effect of silencing ERRα on cell proliferation in uterine endometrial cancer cells, we performed the WST-8 assay. Silencing ERRα significantly inhibited the proliferation of HEC-1A and KLE cells (Figure 3A). Additionally, to investigate the effect of ERRα knockdown on colony formation, we performed colony formation assays. Silencing ERRα significantly reduced HEC-1A colony formation (Figure 3B). Flow cytometry analysis was performed to determine how ERRα knockdown suppressed HEC-1A and KLE cell growth. Silencing ERRα caused the accumulation of cells in the G2/M- (Figure 3C, 3D) and sub-G1-phase (Figure 3C, 3E). To further investigate the G2/M-phase arrest, we performed western blotting analysis. Silencing ERRα resulted in a significant increase of histone H3 Ser-10 (HH3-Ser10) phosphorylation, a representative marker of the mitotic phase, whereas the level of CDC2 and cyclin B1, involved in the G2/M checkpoint, did not change significantly over the same time. This result suggested that the accumulation of cells in the G2/M-phase was responsible for the mitotic arrest (Figure 3F). Additionally, our western blotting analysis showed that silencing ERRα increased the expression of cleaved caspase-3, indicating the initiation of apoptosis. Time course experiment using flow cytometry analysis was performed to clarify the relationship between cell cycle arrest and apoptosis. The accumulation of cells at the G2/M phase was detected 24 h after siRNA transfection in both HEC-1A and KLE cells followed by the recruitment of Sub-G1 cells 36-60 h after the transfection. Our western blot analysis also showed that the increase in HH3-Ser10 phosphorylation was confirmed 24 h after transfection in both cell lines, while the increase of cleaved caspase-3 was detected 48 h after transfection (Figure 3F, 3G), which was consistent with the results obtained by flow cytometry. These results suggest that ERRα loss of function induced cell cycle arrest at the mitotic phase in endometrial cancer cells followed by their apoptosis.

### Effect of ERRα knockdown on the sensitization of HEC-1A cells to paclitaxel

**Figure 4:**
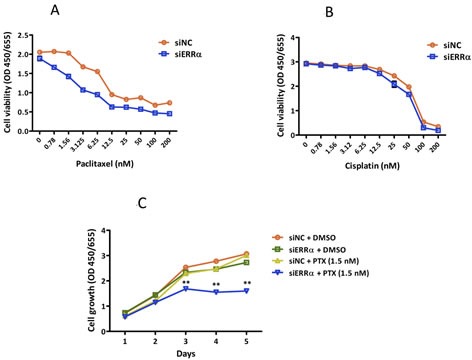
Effect of ERRα knockdown on the sensitivity of endometrial cancer cells to anti-cancer drugs **A.** and **B.**, Sensitivity to anti-cancer drugs using WST-8 assay. Twenty-four hours after siRNA transfection, cells were treated with DMSO alone (control), paclitaxel **A.**, or cisplatin **B.** at the indicated concentrations. After 48 h, cell viability was determined using WST-8 assay. Data represent means ± SEM (*n* = 4). **C.**, Time course analysis of the sensitization effects with 1.5 nM of paclitaxel. Twenty-four hours after siRNA transfection, cells were treated with DMSO alone or 1.5 nM of paclitaxel. Cell viability was then assessed using WST-8 assay every 24 h. Data represent means ± SEM (*n* = 4). 4 4Significant differences are indicated as ** for *P* < 0.01 and * for *P* < 0.05. Each assay was repeated at least three times. siNC, negative control siRNA; siERRα, ERRα siRNA.

Paclitaxel, in combination with cisplatin, is one of the most clinically used anti-cancer drugs for patients with uterine endometrial cancer [23]. To examine the effect of silencing ERRα on the sensitivity to paclitaxel and cisplatin, we treated ERRα knocked down HEC-1A and KLE cells with dimethyl sulfoxide (DMSO) alone (control), paclitaxel, and cisplatin and performed WST-8 proliferation assays. Silencing of ERRα did not sensitize HEC-1A cells to cisplatin, but markedly sensitized the cells to paclitaxel in a dose-dependent manner (IC50; siNC *vs.* siERRα KD = 10.0 *vs.* 6.3 nM) (Figure 4A, 4B). At a concentration of 1.5 nM, paclitaxel alone did not decrease the viability of HEC-1A cells. However, when ERRα was silenced, 1.5 nM paclitaxel significantly inhibited the cell growth of HEC-1A cells in a time-dependent manner (Figure 4C). The significant difference was not found in KLE cells, because KLE cells were too sensitive to paclitaxel resulting in death even at very low experimental concentrations (data not shown).

### Effect of ERRα knockdown on tumor growth and angiogenesis in a mouse xenograft model

**Figure 5:**
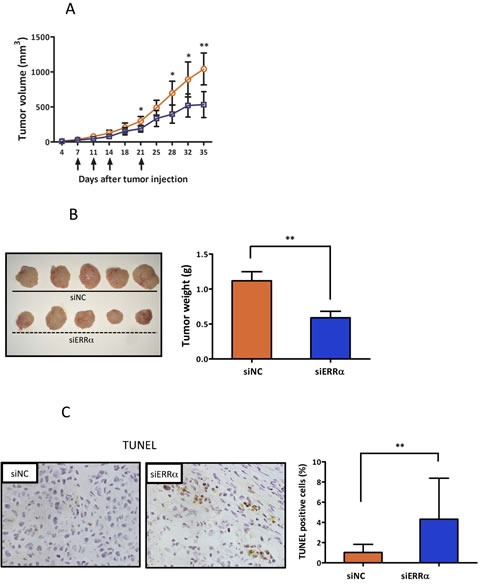

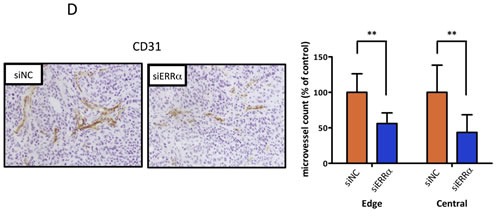
Effect of ERRα knockdown on proliferation and angiogenesis of endometrial cancer cells *in vivo* using a mouse xenograft model **A.**, *In vivo* tumor growth analysis. HEC-1A cells (5 × 106 cells per mouse) were inoculated into the back of the mice by subcutaneous injection. Mice were then locally injected with 1000 pmol of either the control (blue box) or ERRα siRNA (orange circle) at the indicated days (arrows). The tumor volumes were measured twice a week. Data represent means ± SD (*n* = 5). **B.**, Images and weights of excised tumors from each group. **C.**, Apoptotic cells in the tumor sections were detected by TUNEL. The apoptotic index was defined as the percentage of immunopositive cells in 10 high-power fields (× 400). Data represent means ± SD. **D.**, Microvessels were labeled with an anti-CD31 antibody. Microvessels in the densestareas were selected under a low power field (× 40). CD31 immunopositive pixels per microscopic field were counted under a high-power objective (× 200) using ImageJ software. Microvessel density was defined as the percentage of CD31 immunopositive pixels per high-power field (× 200) in 10 different views, and compared in both central and edge areas of the tumor. Data represent means ± SD. Significant differences are indicated as ** for *P* < 0.01 and * for *P* < 0.05. siNC, negative control siRNA; siERRα, ERRα siRNA; TUNEL, terminal deoxynucleotidyl transferase-mediated dUTP nick end labeling.

To further evaluate the effect of ERRα knockdown on endometrial cancer cells *in vivo*, athymic nude mice were subcutaneously inoculated with HEC-1A cells. ERRα knockdown by local injection of the siRNA to the mice significantly suppressed tumor growth when compared to the control (Figure 5A, 5B). We then analyzed apoptosis in the ERRα knockdown tumors using TUNEL assay. Apoptosis was more frequently induced in the tumor sections from mice treated with the ERRα (Figure 5C). Furthermore, to assess the effect of ERRα knockdown on angiogenesis *in vivo*, we analyzed the patterns of micro-vessel density (MVD) using an antibody against CD31. The MVD in tumors from mice treated with the ERRα siRNA was significantly lower than that of tumors from control mice treated with the control siRNA (Figure 5D).

## DISCUSSION

In this study, we demonstrated for the first time that silencing ERRα could inhibit the proliferation of uterine endometrial cancer cells *in vitro* and slow tumor growth *in vivo* in a mouse xenograft model. We also demonstrated that ERRα was highly expressed in the aggressive malignant phenotype and associated with unfavorable clinical outcomes for patients with uterine endometrial cancer. Furthermore, our results indicated that silencing ERRα could inhibit angiogenesis through VEGF and induce mitotic arrest followed by apoptosis. From these findings, we propose that ERRα could be a novel molecular target for the treatment of uterine endometrial cancer.

Previous studies demonstrated that ERRα is expressed in various types of cancer, including hormone-dependent cancers [16, 17]. Suzuki *et al.* first reported that ERRα is expressed in 55% of breast cancer tissues and that the expression was correlated with an increased risk of recurrence and adverse clinical outcome. Other investigators also reported ERRα as one of the negative prognostic factors in human prostate cancer. In this study, we re-confirmed the recent findings that the expression of ERRα in endometrial cancer was correlated with clinic-pathological parameters and showed inverse correlation with disease free survival [24, 25]. These reports also showed a significantly higher expression of ERRα in cancerous tissues than in the normal endometrium. Moreover, we confirmed that ERRα, with the robust co-activator PGC-1α, was expressed in all cell lines regardless of the ERα status. Although this study presents some limitations, including the small sample size, these results indicate that ERRα may be a molecular target for the treatment of uterine endometrial cancer.

We initially focused on a major angiogenic factor, VEGF, because the promoter region of VEGF includes four ERRα binding sites [26]. Our luciferase promoter assay showed that ERRα overexpression activated the VEGF promoter reporter in all endometrial cancer cell lines. Additionally, we demonstrated that ERRα knockdown suppressed the expression and the function of VEGF. Angiogenesis is fundamental for tumor growth/progression to provide nutrients and oxygen necessary for tumor cell proliferation and metastasis [27]. VEGF is a major angiogenic factor even in endometrial cancer and its expression is associated with endometrial cancer prognosis [28, 29]. Recent studies showed that ERRα was co-activated with PGC-1α in response to hypoxia, thereby inducing VEGF expression and angiogenesis in the skeletal muscle [30]. ERRα-dependent regulation of VEGF was reported in breast, prostate, and cervical cancer [31–33]. Our study demonstrates that the ERRα-dependent regulation of VEGF and consequent angiogenesis are important in endometrial cancer.

We next performed loss of function experiments using siRNAs and assessed cell proliferation. Recent studies reported that ERRs are involved in cell proliferation in several cancer cells. However, little information is available on the detailed mechanisms. VEGF contributes not only to angiogenesis, but also to cancer cell proliferation *via* VEGF receptors (VEGF-Rs) [34]. We originally hypothesized that VEGF produced in response to ERRα promoted cancer cell proliferation through autocrine mechanisms. However, we confirmed that VEGF-Rs are not expressed in HEC-1A or KLE cells. Nevertheless, silencing ERRα inhibited cell proliferation in these cell lines. In breast cancer, Stain *et al.* reported that silencing ERRα resulted in a decrease in the growth of xenograft tumor *in vivo*, although it had no effect on the proliferation of estrogen-independent cells *in vitro* [35]. Bianco *et al.* demonstrated that an inverse agonist of ERRα reduced the proliferation of breast and prostate cancer cells by blocking the G1/S transition of the cell cycle [36]. Interestingly, our cell cycle analysis, in uterine endometrial cancer, showed that ERRα knockdown resulted in cell cycle arrest at the G2/M phase. Our western blot analysis showed no significant change in the expression levels of CDC-2, p-CDC2 (Tyr15), and cyclin B1, involved in G2/M check point, while the expression of phospho-histone H3 was significantly increased, indicating the accumulation of mitotic phase cells. These results led us to conclude that the accumulation of cells in G2/M phase was not due to arrest at G2/M transition, but to the metaphase-anaphase transition, during the mitotic phase. Furthermore, our flow cytometry and western blot analysis indicated the induction of apoptosis initiated by the cleavage of caspase-3. Our time course experiments revealed that ERRα knockdown initially induced cell cycle arrest in the mitotic phase followed by apoptosis. Thus, we concluded that the cell cycle arrest at the mitotic phase was caused by ERRα knockdown.

We also demonstrated that silencing ERRα sensitized HEC-1A cells to paclitaxel, but not to cisplatin. Paclitaxel is known to deactivate cells at the mitotic phase by stabilizing microtubule and suppressing their dynamics, whereas cisplatin is not a cell cycle phase specific drug. Recent studies suggested that paclitaxel-induced apoptosis might be correlated with the phosphorylation of Bcl-2 and Bcl-xL [37, 38]. In our study, ERRα knockdown did not increase the phosphorylation of these proteins (Bcl-2 at Ser70, Bcl-xL at Ser62). We also investigated the expression of survivin, which is considered as one of the key regulators of cell division and apoptosis [39], directly associated with polymerized tubulins and contributes to the regulation of microtubule dynamics [40]. A recent study indicated the up-regulation of survivin in paclitaxel resistant endometrial cancer cells when compared with parent cells [41]. However, the expression levels of survivin were not changed in our experiments (data not shown). From these experiments, we speculate that the mechanism of mitotic arrest and apoptosis by ERRα knockdown is different from that by paclitaxel, leading to an increase in the sensitivity to the reagent. An in depth study of the mechanism involved in this effect is warranted.

To evaluate the effect of ERRα knockdown on uterine endometrial cancer cells *in vivo*, we used a mouse xenograft model and determined that silencing ERRα significantly inhibited the growth of subcutaneously transplanted cancer cells. Using TUNEL assay and MVD analysis, we also demonstrated that ERRα knockdown induced apoptosis and suppressed angiogenesis. Taken together, these data confirm the *in vitro* anti-tumor effect of ERRα knockdown on the induction of apoptosis and the inhibition of angiogenesis.

The crosstalk between ERα and ERRα remains controversial. Studies on ERRα initially focused on the correlation with ERα because of significant similarities in their structures, particularly in the DNA binding domain. In breast cancer tissues, one group demonstrated that ERRα expression was not associated with ERα status, while others indicated that the increased level of ERRα was inversely correlated with ERα status. Additionally, the genome wide study conducted by Deblois *et al.* revealed that the functional overlap between ERα and ERRα was quite limited and most of their transcriptional activities were through their specific, but different binding sites in breast cancer [42]. Although the interference between ERα and ERRα in endometrial cancer has not yet been clarified, we previously reported that ERRα competed with ERα for the response element on their target genes, effecting on cell proliferation. In this study, we investigated the functional effect of ERRα inhibition using ERα-independent endometrial cancer cell lines, HEC-1A and KLE cells. We also evaluated the effect of silencing ERRα using ER-α-dependent Ishikawa cells. However, the suppressive effect on angiogenesis and cell proliferation was not observed (data not shown). These results indicate the existence of a complex crosstalk between ERα and ERRα in endometrial cancer.

In this study, we demonstrated that ERRα could be a potential molecular target in ER-negative endometrial cancer cells. Further studies are warranted to strengthen our current findings and to analyze the functional crosstalk between ERα and ERRα. These results can help in the development of new hormonal and molecular targeted therapy for endometrial cancer.

## MATERIALS AND METHODS

### Reagents

Paclitaxel was purchased from Sigma-Aldrich (St. Louis, MO, USA). Cisplatin was purchased from Wako Pure Chemicals (Osaka, JAPAN).

### Antibodies

Rabbit polyclonal anti-phosphorylated-CDC2 (Tyr15) (#9111), anti-CDC2 (#9112), anti-cleaved caspase 3 (#9661), anti-caspase 3 (#9662), rabbit monoclonal anti-phosphorylated-Histone H3 (ser10) (#9706), anti-GAPDH (#2118), mouse monoclonal anti-cyclin B1 antibodies (#4135) were purchased from Cell Signaling Technology (Danvers, MA, USA). Mouse monoclonal anti-ERRα antibody (sc-65715) was purchased from Santa Cruz Biotechnology (Santa Cruz, CA, USA). Rabbit polyclonal anti-CD31 antibody (ab28364) was purchased from Abcam (Cambridge, UK). All antibodies were used at the concentration recommended by the manufacturers.

### Patients, specimen collection, and immunohistochemistry

Specimens from 53 patients with uterine endometrial cancer, who underwent primary operation at the Department of Obstetrics and Gynecology, Kyoto Prefectural University of Medicine (Kyoto, Japan) between 2006 and 2010, were evaluated. These patients did not receive any chemotherapy or radiation therapy before surgery. The research protocol was approved by the Institutional Review Board and an informed consent was obtained from all patients prior to the beginning of the study. Immunohistochemical staining was performed as previously described [43]. Furthermore, the expression level of ERRα was assessed by H-score, a commonly used method to measure the strength of ER- and ERR-staining, which semi-quantitatively evaluates both the intensity and the percentage of cells stained at each intensity [24]. Intensities were scored as 0 (no staining), 1 (weak staining), 2 (moderate staining), 3 (strong staining) as shown in Supplementary Figure S1. The H-score was calculated by the following algorithm: H-score = Σ(i+1) × Pi (i and Pi represent intensity and percentage of cells at each intensity). We also used the quantification method, which was appropriate for analyzing the relation of H-score to survival rate, separating H-score into 4 groups: 1-50, 51-100, 101-200, and > 200 [44].

### Cell lines and culture

Human endometrial cancer cell lines, HEC-1A, KLE, and RL95-2 cell lines were purchased from the American Type Culture Collection (Manassas, VA, USA). Ishikawa and SNGII cell lines were provided by the Cell Resource Center for Biomedical Research (Institute of Development, Aging and Cancer Tohoku University, Sendai, Japan). HEC-1A cells were cultured in McCoy's 5A medium (HyClone Laboratories, South Logan, UT, USA), KLE and RL95-2 cells were maintained in Dulbecco's Modified Eagle Medium (DMEM)/F12 (Nacalai Tesque, Kyoto, Japan), SNGII cells were cultured in Ham's F12, and Ishikawa cells were cultured in Modified Eagle Medium (MEM) (Nacalai Tesque). Each medium was supplemented with 10% fetal bovine serum (FBS) (Biowest, Nuaille, France) and penicillin-streptomycin (Nacalai Tesque). All cells were cultured at 37°C in a humidified 5% CO_2_ atmosphere.

### Plasmid construction

#### Mammalian expression vectors

The ERRα and PGC-1α expression plasmids were constructed by inserting the full-length human ERRα and PGC-1α gene (NM_004451 and NM_013261), amplified from pSG5-ERRα and -PGC-1α, kindly provided by Prof. Shiuan Chen, into the pcDNA3.1-empty plasmid (Invitrogen, Carlsbad, CA, USA) using the HindIII and XhoI sites. The pcDNA3.1-empty vector was used as a control.

#### Reporter constructs

Luciferase reporter plasmids, pGL3-VEGF promoter constructs containing four copies of ERRE site, (5′-AGGTCA-3′), were kindly provided by Prof. Salman Hyder (University of Missouri, Columbia, MO, USA). pGL4.74 vector (Promega, Madison, WI, USA) was used to normalize the luciferase activities.

#### Transient transfection and luciferase reporter assay

Twenty-four to 72 h after transfection using Lipofectamine LTX (Invitrogen), the luciferase reporter assay was performed with the Dual-Glo Luciferase Assay System (Promega) according to the manufacturer's protocol. Briefly, cancer cells were seeded in 24-well plates and incubated overnight. Cells were co-transfected with pGL3-VEGF promoter reporter plasmid, expression plasmid (pcDNA3.1- empty and/or - ERRα and/or - PGC-1α), and pGL4.74 vector as transfection control. After 24 h of incubation, cells were lysed and assayed. The luminescence was measured with Glomax 20/20 luminometer (Promega).

#### *In vitro* small interfering (si) RNA transfection

Small interfering RNAs (siRNA) for ERRα (s4829, s4830, and s4831) and negative control siRNA (control #1) were Silencer Select siRNAs purchased from Ambion (Austin, TX, USA), the specificity and knockdown effect of which have already been confirmed and guaranteed by the Manufacturers. The siRNA transfection experiments were performed using Lipofectamine RNAiMAX (Invitrogen) according to the manufacturer's protocol. The knockdown effects of the siRNAs were confirmed by using real-time PCR and western blotting analysis. For the following experiments, we used siRNA for ERRα (s4831) at a final concentration of 5 nM because it presented the strongest knockdown activity.

### RNA extraction and quantitative real-time PCR

Total RNA (1 μg) was extracted from cultured cells using RNeasy Mini kit (QIAGEN, Venlo, Netherlands) and then used to synthesize cDNA with ReverTra Ace qPCR RT kit (Toyobo, Osaka, Japan). Quantitative real-time PCR was performed using CFX Connect Real-time PCR Detection System (Bio-Rad, Hercules, CA, USA) with Thunderbird SYBR qPCR Mix (Toyobo) and primers for target genes. The following primers, *ERRα* 5′-ATGGTGTGGCATCCTGTGAG-3 (forward) and 5′-TGGTGATCTCACACTCGTTGG-3′ (reverse), Glyceraldehyde 3-phosphate dehydrogenase (*GAPDH*) 5′-GCACCGTCAAGGCTGAGAAC-3′ (forward) and 5′- ATGGTGGTGAAGACGCCAGT-3′ (reverse), and *VEGF* 5′-CGTGATGATTCTGCCCTCCT-3′ (forward) and 5′-CCTTGCCTTGCTGCTCTACC-3′ (reverse) were designed with primer 3 plus software and purchased from Invitrogen. The target gene mRNA level was quantified using the comparative method (ΔΔCT method) and normalized to *GAPDH* expression.

### ELISA

The amount of human VEGF protein was determined using a Quantikine ELISA kit (R&D systems, Minneapolis, MN, USA) according to the manufacturer's protocol. Briefly, cells were seeded in 10 cm dish and RNA interference experiment was performed after 24 h. After 72 h, the cell culture medium was collected and centrifuged at 1,500 rpm for 5 min to remove any particles. The supernatants were frozen at 80°C until used. Human recombinant VEGF165 provided in the Quantikine ELISA kit was used as a standard.

### Cell viability assay

Cells were seeded in 96-well plates with normal growth medium. RNA interference was performed after 24 h. Cells were cultured, treated, and cell viability was examined every day by the 2-(2-methoxy-4-nitrophenyl)-3-(4-nitrophenyl)-5-(2,4-disulfophenyl)-2H-tetrazolium, a monosodium salt (WST-8) assay (Nacalai Tesque). Each experiment was performed three times using 4 replicates.

### HUVEC proliferation assay

Cancer cells were seeded in 10 cm dish and incubated for 24 h. Subsequently, the cells were transfected with siRNA. After 72 h, the cultured medium was collected and centrifuged at 1,500 rpm for 5 min to remove any particles. The supernatants were frozen at 80°C. Human umbilical vein endothelial cells (HUVEC) were seeded in 96-well plates. The culture medium was then replaced by the collected supernatants every 24 h. HUVEC proliferation was assessed by using the WST-8 assay every day.

### HUVEC migration and invasion assay

Migration and invasion assays were performed using uncoated and Matrigel-coated double-chamber systems (BD BioCoat^TM^ Matrigel^TM^ Invasion Chamber, BD Biosciences, Bedford, MA, USA) as previously described [43]. Briefly, cancer cells were seeded into 24-well lower chamber filled with 10% FBS contained medium and, after 24 h, cells were transfected with siRNAs. After 72 h incubation, HUVEC were then seeded into upper chamber (uncoated or Matrigel-coated inserts) filled with 1% FBS containing medium. After 12 h, cells that migrated and invaded onto the lower side of the inserts were fixed and stained with Diff-Quick Kit (Sysmex, Kobe, Japan). The number of stained cells was counted in five fields using a microscope (200×).

### Colony formation assay

Cells were seeded in 6 cm dishes and transfected with siRNAs. After 24 h, cells were harvested by trypsinization and reseeded at a density of 200 cells in each well of 6-well plates. Cells were then incubated for 14 days and stained with crystal violet.

### Flow cytometry analysis

Cells were seeded in 6-well plates and RNA interference was performed. Cells were permeabilized with 0.1% Triton-X100 and the nuclei were then stained with propidium iodide (PI) after 24-48 h. The DNA content was measured using a FACS Caliber cytometer (BD biosciences) and analyzed with the ModFit LT (Verity Software, Topsham, ME, USA) and Cell Quest software package (BD biosciences).

### Western blot analysis

Cell protein extracts were collected using Radioimmunoprecipitation (RIPA) buffer (Nacalai Tesque) and then mixed with SDS sample buffer (62.5 mM Tris-HCL pH 6.8, 10% glycerol, 1% SDS, 0.1% 2-mercaptoethanol, 1 mM phenylmethylsulfonyl fluoride) and heated for 15 min at 65°C. The lysates were loaded onto polyacrylamide gels, subjected to electrophoresis, and transferred to a polyvinylidene difluoride membrane. The blots were blocked in blocking buffer (5% skim milk/TBS-Tween) for 30 min at room temperature and incubated with appropriate primary antibody in blocking buffer overnight at 4°C. The blots were incubated with the appropriate secondary antibody in blocking buffer for 1 h at room temperature. The signal was detected with Chemi-Lumi One (Nacalai Tesque) and ChemiDoc XRS+ system (Bio-Rad).

### *In vivo* animal study

Female BALB/c mice (5 weeks of age) were purchased from Shimizu Co., Ltd. (Kyoto, Japan). For the *in vivo* study, siRNAs and the *in vivo* transfection kit, AteloGene Local Use, were purchased from Koken (Tokyo, Japan) [45]. The sequence of ERRα siRNA used *in vivo* was the same as that of the siRNA used *in vitro*. HEC-1A cells (5 × 10^6^ cells per mouse) were inoculated into the back of the mice by subcutaneous injection. The tumor volume was calculated using the following formula: 1/2 × (length) × (width)^2^. After the establishment of palpable tumors (approximately 50 mm^3^), mice were locally injected with 1000 pmol of either control or ERRα siRNAs with AteloGene Local Use (total 0.1 mL) on day 7, 11, 14, and 21 after tumor injection, according to the manufacturer's protocol. Mice were sequentially monitored twice a week for 5 weeks by measuring the tumor volume and body weight. On day 28, the tumors were excised from the euthanized mice, embedded in Tissue-Tek compound (Sakura Finetek, Tokyo, Japan), and frozen in liquid nitrogen. All experiments and procedures were approved by the Institutional Care Use Committee and performed in accordance with guidelines.

### *In vivo* analysis of micro-vessel density (MVD)

To quantify tumor angiogenesis, MVD was quantified. First, micro-vessels were immunostained with anti-CD31 antibody as follows: Tumor frozen sections were sliced to 6-μm thickness, dried for a few hours at room temperature, and fixed in 10% formalin. Sections were washed with PBS thrice and the endogenous peroxidase activity was blocked with 0.3% hydrogen peroxide in methanol for 20 min at room temperature. Sections were then blocked for 30 min with 2% normal swine serum (VECTOR Laboratories, Brussels, Belgium) in PBS. Incubation with primary polyclonal rabbit anti-CD31 antibody (1:50) was performed at 4°C overnight. Slides were then incubated with a biotinylated secondary antibody (VECTOR Laboratories) (1:200) for 30 min at room temperature. Slides were incubated with VECTASTAIN Elite ABC Kit (VECTOR Laboratories). 3,3′-Diaminobenzidine (DAB) staining was performed (DAB TRIS Tablet, Muto Pure Chemicals, Tokyo, Japan) to detect CD31 and the nuclei were counterstained with Mayer's hematoxylin (Muto Pure Chemicals). MVD was then analyzed as follows: Slides were scanned under low power field (×40) to select micro-vessels in the densest areas. CD31 immunopositive pixels per microscopic field were counted under high power objective (×200) using Image J software (NIH, Bethesda, MD, USA). MVD was quantified as the percentage of CD31 immunopositive pixels per high-power field in 10 views.

### *In vivo* analysis of apoptosis

Apoptotic cells in the 6 μm thick frozen sections were detected by a terminal deoxynucleotidyl transferase-mediated dUTP nick end labeling (TUNEL) with the DeadEnd colorimetric Apoptosis Detection System (Promega), according to the manufacturer's protocol. The apoptotic index was defined as the percentage of immunopositive cells in 10 high-power fields (×400).

### Statistical analysis

The relation of the H-score to clinico-pathologic factors (clinical stage, histological type, and myometrial invasion) was analyzed using the Kruskal-Wallis H-test and the Mann-Whitney U-test with Bonferroni correction was used as a post hoc test. Comparisons of the means and standard error of data between two groups were performed using the Student's t test. Disease-free survival was estimated according to the Kaplan-Meier method and log-rank test was used to calculate the statistical significance. *P* values < 0.05 were considered statistically significant.

## SUPPLEMENTARY MATERIAL FIGURE


