# Supplementary material for: Anti-tumor effect of estrogen-related receptor alpha knockdown on uterine endometrial cancer
Source: Oncotarget. 2016 May 3;7(23):34131–48. doi: 10.18632/oncotarget.9151 (PMC5085142; doi:10.18632/oncotarget.9151)
Supplement: Supplementary file 1 [file oncotarget-07-34131-s001.pdf]

Anti-tumor effect of estrogen-related receptor alpha knockdown on uterine endometrial cancer

Supplementary Material

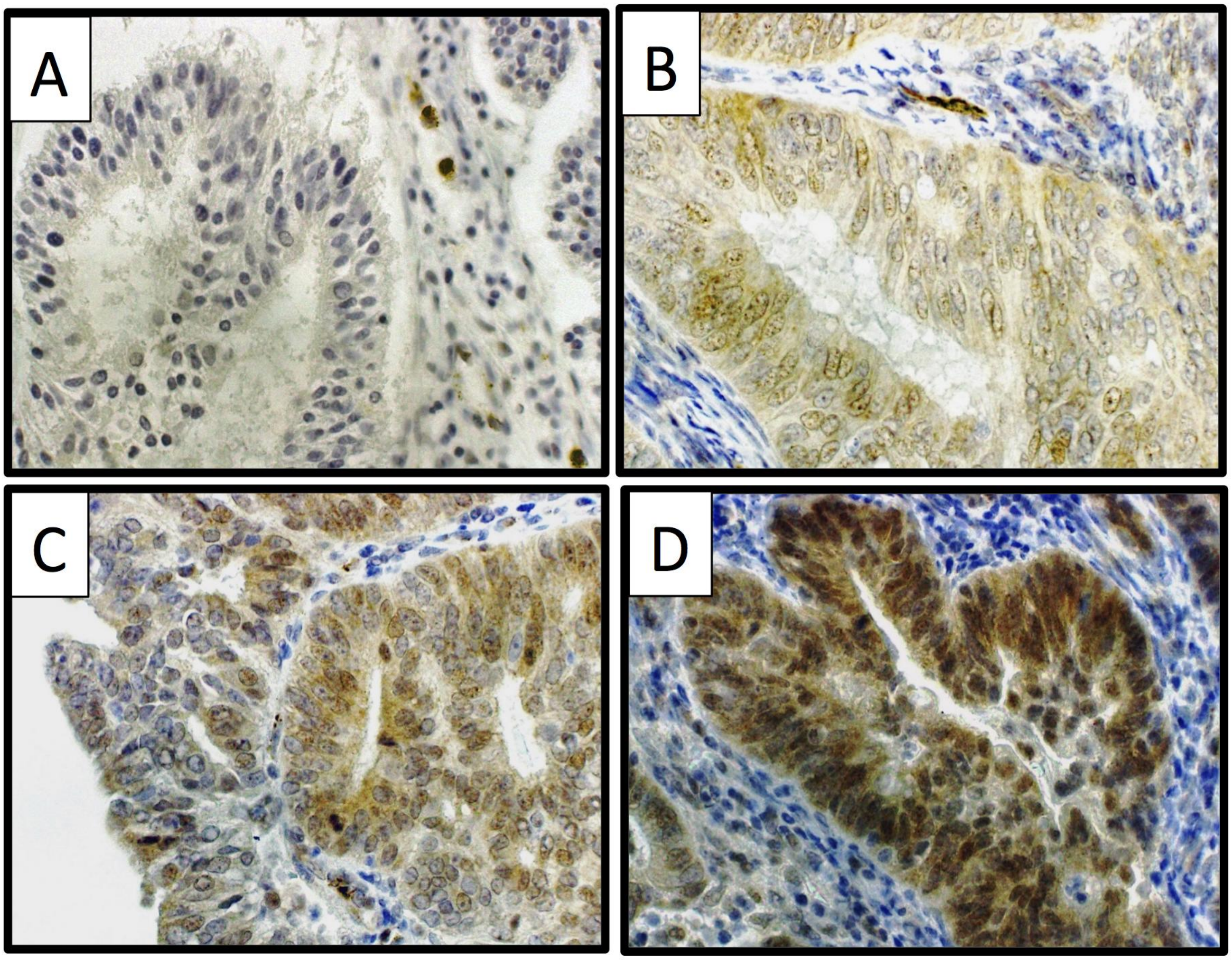

Supplementary Figure S1

The intensity of the immunohistochemical staining for ERRα was scored as 0 (no staining) (A), 1 (weak staining) (B), 2 (moderate staining) (C), and 3 (strong staining) (D).
